# Transcriptional repression of the oncofetal *LIN28B* gene by the transcription factor SOX6

**DOI:** 10.1038/s41598-024-60438-3

**Published:** 2024-05-04

**Authors:** Valentina Pastori, Gianluca Zambanini, Elisabetta Citterio, Tamina Weiss, Yukio Nakamura, Claudio Cantù, Antonella Ellena Ronchi

**Affiliations:** 1 Dipartimento di Biotecnologie e Bioscienze, Università degli Studi di Milano-Bicocca, Milan, Italy; 2 Wallenberg Centre for Molecular Medicine, Linköping University, Linköping, Sweden; 3 Division of Molecular Medicine and Virology, Department of Biomedical and Clinical Sciences, Faculty of Medicine and Health Sciences, Linköping University, Linköping, Sweden; 4 Max-Planck-Institut für molekulare Genetik, Berlin, Germany; 5 RIKEN BioResource Research Center, Tsukuba, Ibaraki Japan

**Keywords:** Developmental biology, Genetics, Oncology

## Abstract

The identification of regulatory networks contributing to fetal/adult gene expression switches is a major challenge in developmental biology and key to understand the aberrant proliferation of cancer cells, which often reactivate fetal oncogenes. One key example is represented by the developmental gene *LIN28B*, whose aberrant reactivation in adult tissues promotes tumor initiation and progression. Despite the prominent role of LIN28B in development and cancer, the mechanisms of its transcriptional regulation are largely unknown. Here, by using quantitative RT-PCR and single cell RNA sequencing data, we show that in erythropoiesis the expression of the transcription factor *SOX6* matched a sharp decline of *LIN28B* mRNA during human embryo/fetal to adult globin switching. SOX6 overexpression repressed *LIN28B* not only in a panel of fetal-like erythroid cells (K562, HEL and HUDEP1; ≈92% *p *< 0.0001, 54% *p *= 0.0009 and ≈60% *p *< 0.0001 reduction, respectively), but also in hepatoblastoma HepG2 and neuroblastoma SH-SY5H cells (≈99% *p *< 0.0001 and ≈59% *p *< 0.0001 reduction, respectively). SOX6-mediated repression caused downregulation of the LIN28B/Let-7 targets, including *MYC* and *IGF2BP1*, and rapidly blocks cell proliferation. Mechanistically, *Lin28B* repression is accompanied by SOX6 physical binding within its locus, suggesting a direct mechanism of *LIN28B* downregulation that might contribute to the fetal/adult erythropoietic transition and restrict cancer proliferation.

## Introduction

Development is dictated by the timely integration of a complex network of genetic regulatory circuits. The identification of these fetal/adult switches is key to understand not only the developmental process but also the aberrant proliferation of cancer cells, which very often rely on the expression of fetal oncoproteins to sustain their proliferation^1^.

***LIN28B*** is an oncofetal gene whose expression is preferentially confined to early developmental stages in various tissues. Originally identified in C. elegans, where it controls the timing of larval development^2^, LIN28 is a RNA-binding protein encoded in mammalians by two genes, Lin28A and Lin28B^3,4^. In humans, *LIN28* has emerged as a Quantitative Trait Locus (QTL), influencing the timing of developmental processes,^5–8^ and has been implicated in cell reprogramming^9^, tissue repair^10^ and cancer, where it is upregulated in a large number of human primary cancers and tumor cell lines^11–18^.

A major role of LIN28B is to block the processing from precursor to mature miRNAs of Let-7 family, which promote differentiation by downregulating growth-promoting genes, including *c-MYC*, *RAS*, *IGF2BP1-3* and *HMGA2*^19^. This evidence suggests that the oncogenic role of LIN28B involves the de-repression of pro-proliferative Let-7 targets.

In hematopoiesis, LIN28 expression is confined to fetal HSPCs cells. Its ectopic expression in adult cells promotes a fetal-like phenotype, characterized by active self-renewal and fetal-like lymphoid and myelo-erythroid differentiation^20–23^. LIN28B expression in hematopoietic adult cells represses Let-7^24^, the most upregulated miRNAs family in adult versus fetal erythroblasts^25^. Interestingly, LIN28B overexpression is observed in specific subtypes of pediatric leukemia^26^, including juvenile myelomonocytic leukemias of fetal origin (JMML), characterized by high fetal hemoglobin levels and considered to arise from a stem-progenitor cell^27^, possibly of fetal origin^24^^,^^28^^,^^29^.

**SOX6** is a member of the HMG box family of Transcription Factors, preferentially expressed in adult cells^30^. In hematopoiesis, SOX6 is expressed in quiescent LT-HSC^31^ and it acts as a tumor suppressor in chronic myeloid leukemia stem cells (CML-LSC)^32^. In erythropoiesis, it is expressed in adult cells, where it promotes differentiation^33,34^ and cooperates with the BCL11A-XL in the silencing of fetal γ-globin^35^.

SOX6 downregulation is observed in different human cancers, including acute myeloid leukemias^36^, Its low expression often correlates with poor prognosis. Restoring SOX6 expression mitigates the hyper-proliferative phenotype, indicating that it can act as tumor suppressor^37–41^.

Here we show that SOX6 represses *LIN28B* in different human fetal-like erythroid cell types and in myeloid leukemia cell lines. The repression of *LIN28B* upon SOX6 transduction results in the repression of Let-7 targets, including *c-MYC*, and in arrested cell growth.

We find that the repression of *LIN28B* by SOX6 also occurs in tumor cell types representative of other tissues, such as SH-SY5Y neuroblastoma and HepG2 hepatoblastoma cells, where *LIN28B* is highly expressed^13,14^. In all the contexts tested, the repression of *LIN28B* upon SOX6 overexpression invariantly results in decreased cell growth and proliferation. Finally, we identify SOX6 consensus binding sites within the *LIN28B* genomic locus. ChIP-qPCR and CUT&RUN experimentally validate SOX6 physical occupancy of these sites, supporting a direct transcriptional repression of *LIN28B* by SOX6.

Overall, our data reveal a mechanism by which SOX6 can act as a tumor suppressor in *LIN28B*-positive cancer cells.

## Materials and methods

### Cell and cell cultures

Cell lines, culture media, supplements and conditions are listed in Supplementary table 1.

CD1 mice were housed in our animal facility with 12 h light and dark cycles and free access to water and chow. Females 8–14 week-old were mated with males from the same strain and inspected daily for vaginal plugs. The appearance of the vaginal plug was designated as day 0.5 of pregnancy. At days 11.5, 12.5 and 13.5 pregnant females were sacrificed by cervical dislocation. N = 3–5 females were sacrificed for each time point and embryos from the same litter were pooled for subsequent analysis. Bone Marrow (BM) was harvested from the sacrificed pregnant females. Mouse experiments were conducted under the approval of the Italian Ministry of Health (protocol number 358-2016/PR), in accordance with European Union (86/609/EEC) and ARRIVE guidelines.

### Data analysis from publicly available single-cell RNA sequencing datasets

The single-cell RNA sequencing (scRNAseq) datasets, encompassing human fetal liver and yolk sac from ref Popescu et al.^42^, human neuroblastoma from Kildisiute et al.^43^, and human hepatocellular carcinoma from Lu et al.^44^, were publicly available and underwent preprocessing as outlined in the Table 1. Subsequently, Anndata objects were analyzed using Python packages Scanpy^45^ (v.1.9.5) and Anndata (https://github.com/scverse/anndata) (v.0.9.2), inspired by Seurat^46^, to visualize the expression of genes of interest. Cluster assignment of cell types was achieved utilizing cell labels and marker genes sourced from the respective papers. For the yolk sac dataset, neighborhood graphs were constructed with 10 neighbors and 40 principal components (PCs), followed by dimension reduction to two dimensions using UMAP as recommended in the Scanpy clustering pipeline.

**Table 1:** Single-cell RNA sequencing data sets and processing.

	Fetal liver	Yolk sac	Neuroblastoma	Hepatocellular carcinoma
Species	Human	Human	Human	Human
Number of subjects	14	3	16	10
Cell count	113,063	10,071	13,281	16,498
Tissue /design	Fetal liver 7–17 PCW	4–6 PCW	Mainly pre-treated tumors, viable tumor areas	Tumor and adjacent liver, primary and relapsed tumor
scRNAseq Kit	10 × 3’ v2		CEL-seq2	10 × 3’ v2
Preprocessing	Cell ranger alignment on GRCh38 (STAR), Cells filtered for > 200 detected genes and total mtCount < 20%; Genes filtered for expressed in > 3 cells		Cell ranger alignment on GRCh38 (STAR), Cells filtered for > 200 detected genes and > 500 UMIs and total mtCount < 20%; Genes filtered for not mtGenes and not hspGenes	Cell Ranger alignment on GRCh38, Cells filtered for > 200 detected UMIs and > 200/ < 8000 detected genes and total mtCount < 10%
Normalization	By sequencing depth scale to 10,000 counts (NormalizeData, LogNormalize method), data feature scaling, variable gene detection, PCA, Louvain graph-based clustering with a resolution of 30 with standard parameters (fetal liver only, Seurat)		By sequencing depth scaled to 10 000 counts (NormalizeData, LogNormalize method), Scaling, variable gene detection (most 2000), PCA of 2000 most variable genes, 50 first PCs were used to calculate a UMAP (resolution parameter 1) (Seurat)	Seurat standard normalization pipeline, Shared-nearest neighbor clustering obtained a final of 53 clusters that were used to calculate a UMAP (Resolution parameter 3) (Seurat)
Download source	https://developmental.cellatlas.io/fetal-liver		http://neuroblastomacellatlas.org	http://omic.tech/scrna-hcc/
Download format	Anndata .h5ad format containing count matrix and metadata		Anndata .h5ad format count matrix and metadata	Anndata .h5ad format count matrix and metadata
Reference	Popescu et al.^42^ (Nature)		Kildisiute et al*.*^43^ (Science Advances)	Lu et al*.*^44^ (Nature Communications)
Raw data	E-MTAB-7407 (Array Express)		EGAD00001008345	EGAC00001001616

### RNA isolation and real time PCR

Total RNA from ≥ 10^5^ cells were purified with TRIzol Reagent (Euroclone) and retrotranscribed (High Capacity cDNA Reverse Transcription Kit, Applied Biosystem). RT-PCR analysis was performed using StepOne (Thermofisher). Specific PCR product accumulation was monitored by using SsoAdvanced™ Universal SYBR^®^ Green Supermix (Bio-Rad) fuorescent dye in 12 μl reaction volume. Dissociation curves confirmed the homogeneity of PCR products. Primers, designed to amplify 150 to 300bp amplicons, are listed in the Supplementary table 3. Each experiment was done in three biological replicates and cDNAs from each replicate were analysed in technical triplicates.

### Protein extracts

For whole protein extracts, cells were resuspended in RIPA buffer (20 mM Tris HCl pH 7.4, 137 mM NaCl, 10% glycerol, 0.1% SDS, 0.5% deoxycolate, 1% Triton X-100, 2mM EDTA and proteases inhibitor cocktail). Lysis was performed for 30 min in ice and, after centrifugation (15 min at 16,000×g at 4 °C) the supernatant was collected and analysed.

### Immunoblotting

Protein extracts were resolved by SDS/PAGE in a 10% acrylamide gel and blotted onto Hybond-ECL Nitrocellulose membrane (GE healthcare) at 400 mA for 90 min at 4 °C (Biorad Transblot apparatus). Membranes were blocked for 1 h at RT with milk 5% in TBS-T (Tris Buffered Saline, pH 7.6 and 0,1% Tween20) and incubated with the appropriate primary antibody diluted in milk 5% TBS-T overnight at 4 °C. Membranes were washed in TBS-T and incubated with the appropriate HRP-conjugated secondary antibody (in milk 5% TBS-T) for 1 h at room temperature. Antibodies binding was detected by ECL (Millipore). The blots were cut prior to hybridisation with the indicated antibodies. Full size gels are shown in Supplementary Fig. 10.

### Lentiviral particles production

The Sox6 murine cDNA was cloned in frame with a FLAG epitope immediately upstream to the IRES-eGFP cassette into the CSI vector, as in^34^. Packaging HEK-293T cells were transfected with the SOX6 expression vector (or the corresponding empty control Vector, EV) and with the psPAX2 and pMD-VSVG packaging vectors. 72 h upon transfection, viral pseudoparticles were concentrated by centrifugation at 4 °C. The viral pellet was resuspended in 1X PBS and aliquoted at − 80 °C. 72h upon lentiviral transduction (MOI ≥ 25) GFP + cells were scored by Flow Cytometry (FC) (CytoFlex, Beckman Coulter). For HUDEP1 and HepG2 cells where GFP positivity was about 60–70%, cells were sorted (BD FACSMelody, BD Biosciences) before further analysis. All experiments were performed on cells ≥ 90% GFP + (Supplementary Fig. 1).

### CUT&RUN analysis

The detailed protocol for CUT&RUN on HUDEP1 overexpressing SOX6-Flag and for data analysis are detailed in the Supplementary file material. Sox6 peaks are listed in suppl. table 4. The CUT&RUN datasets (raw and processed files) have been deposited at ArrayExpress (https://www.ebi.ac.uk/arrayexpress/) under accession number E-MTAB-12800.

### Chromatin IP

HEL cells overexpressing SOX6 were fixed with 0.4% formaldehyde for 10 min at RT. Chromatin was sonicated (Bioruptor^®^, Diagenode) to the size of ≈500bp. DNA immunoprecipitation was obtained by incubation with the appropriate antibodies and subsequent isolation with protein A-agarose beads (Upstate). Immunoprecipitated DNA fragments were analysed by qPCR for the regions of interest (primers are listed in Supplementary Table 3). Full size gels are shown in Supplementary Fig. 9.

### Primers, antibodies and reagents

Antibodies, reagents and primers are listed in the Supplementary Tables 2 and 3.

### Statistical analysis

All experiments were performed on a least of n ≥ 3 biological replicates, each analysed in technical triplicates. For Western Blots, statistics was calculated on densitometry values from 3 independent experiments obtained by using ImageJ software. Statistical analysis was performed by using GraphPad Prism version 8.0.0 for Windows, GraphPad Software (www.graphpad.com). Data were analysed by using a two-tailed unpaired t-test and are expressed as mean ± SEM (standard error of mean). In Figures, error bars represent SEM and p-values are represented as follows: *p < 0.05; **p < 0.01; ***p < 0.001. The numerical values are in Figure legends.

Figures were prepared in compliance with the Scientific Reports digital image and integrity policies.

## Results

### *Sox6* and *Lin28B* show opposite expression profiles during the fetal to adult transition in erythroid cells

**Figure 1:**
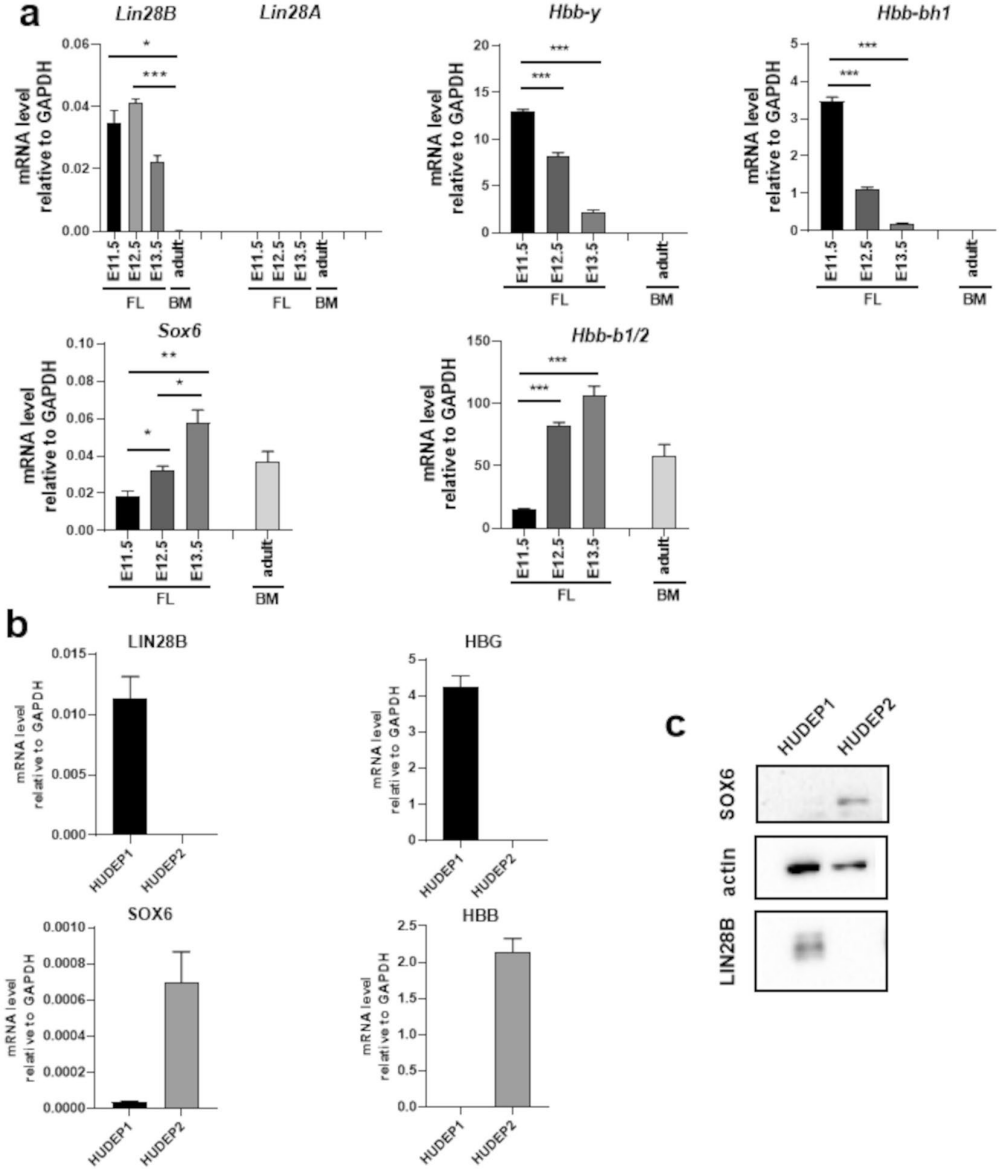
Expression levels of *LIN28* and *SOX6* in erythropoiesis during development. (**a**) RT-qPCR: *Sox6*, *Lin28B* and globins expression relative to *GAPDH* in E11.5-E13.5 mouse fetal liver (FL) and in adult bone marrow (BM). The decline in *Lin28B* and the opposite increase in *Sox6* parallel the transition (hemoglobin switching) from embryo/fetal (*Hbb-y* and *Hbb-bh1*) to adult (*Hbb-b1/2*) mouse globins. In the Figure, * = *p* < 0.05; ** = *p* < 0.01; *** = *p* < 0.001. *Lin28B* and *Sox6* fold changes calculated on mean values: *Lin28B* fold reduction E12.5-E13.5 ≈45% *p* = 0.0003, *Sox6* fold increase E12.5-E13.5 ≈45% *p* = 0.012. (**b**) RT-qPCR and Western Blot showing the opposite expression of *LIN28B* and *SOX6* and human fetal γ (*HBG*) and adult β (*HBB*) globins in human fetal-like HUDEP1 and adult HUDEP2 cells. (**c**) Western blot showing the relative protein levels.

Erythropoiesis represents a model system of developmental dynamics. In particular, in the mouse fetal liver (FL), the E11.5-E13.5 timeframe corresponds to the transition from embryo/fetal to adult erythropoiesis, marked by the switching form embryo/fetal to adult globin genes expression (Fig. 1a). RT-qPCR shows that, within this developmental window, the expression of *Sox6* and *Lin28B* inversely correlate: the highest expression of *Sox6* at E13.5 coincides with the drop in *Lin28B* and with the establishment of the adult pattern of globin expression. Consistently, in adult bone marrow (BM) erythropoiesis where *Sox6* expression persists throughout adult life, *Lin28B* is absent (Fig. 1a). *Lin28B* and *Sox6* expression profiles, both at the mRNA and protein level, also inversely correlate in human HUDEP1 and HUDEP2 cells^47^, representative of embryo/fetal and adult erythropoiesis, respectively (Fig. 1b and c). The *Lin28B* paralog, *Lin28A*, is not expressed (Fig. 1a).

### SOX6 and LIN28B/Let-7 downstream genes are expressed in non-overlapping cell populations during human embryo/fetal hematopoiesis

**Figure 2:**
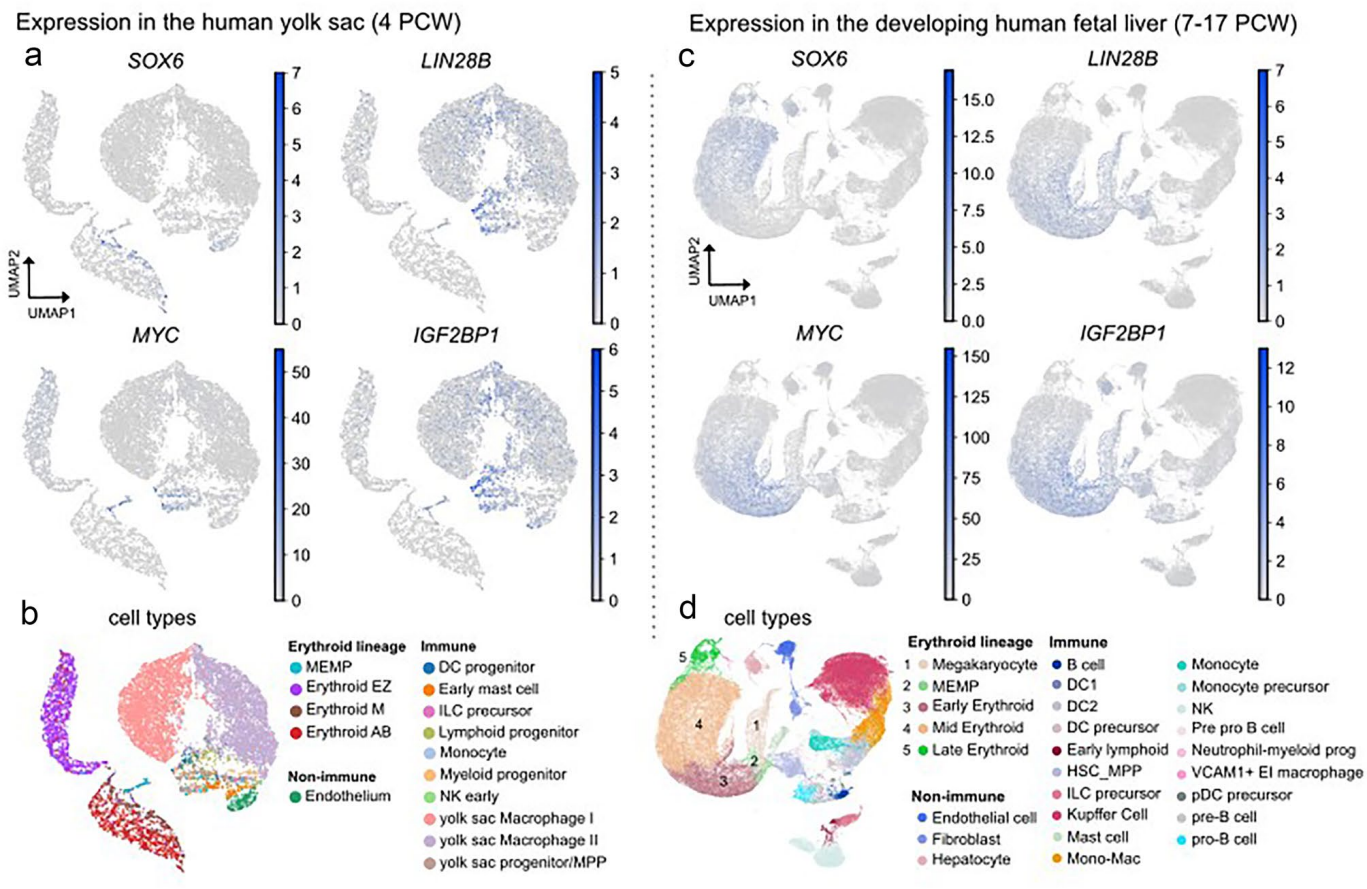
Single-cell RNA expression of the SOX6-LIN28B/Let-7 axis in human fetal hematopoiesis. (**a**) Normalized expression of *SOX6*, *LIN28B*, as well as the downstream genes *MYC*, and *IGF2BP1* in human yolk sac cells shown as UMAP. (**b**) 15 UMAP clusters are associated with cell types according to the expression of distinct marker genes from Popescu et al.^42^. Dataset contains 10,071 yolk sac cells derived from 3 individuals at the age of 4–6 PCW. (**c**) Normalized expression of *SOX6*, *LIN28B*, as well as the downstream genes *MYC*, and *IGF2BP1* in human fetal liver cells shown as UMAP (Uniform Manifold Approximation and Projection) plots. (**d**) 27 UMAP clusters are associated with cell types according to the expression of distinct marker genes. Dataset contains 113,063 fetal liver cells derived from 16 individuals at the age of 7–17 PCW. *PCW*, post conception week; *MEMP*, megakaryocyte-erythroid progenitors; *DC*, dendritic cells; *HSC*-*MPP*, hematopoietic stem cells and multipotent progenitors; *ILS*, induced leucocyte stem; *Erythroid EZ*, early (HGE expressing) erythroid; *Erythoid M*, mid erythroid; *Erythroid AB*, late (HGA and HGB expressing) erythroid.

To investigate the relation between *SOX6* and *LIN28B* expression in human early development at a single-cell resolution, we analyzed single-cell RNA sequencing (scRNA-seq) datasets obtained from yolk-sac (4 PCW, post conception weeks) and fetal liver (7 to 17 PCW) cells^42^. The integrated data analysis at these stages shows that the expression of *SOX6* and *LIN28B* is confined to distinct cell populations (Fig. 2 and Supplementary Fig. 2): whereas *LIN28B* and the LIN28B/Let-7 downstream targets *MYC* and *IGF2BP1* are prominently expressed in early erythroid progenitors (MEMP), *SOX6* raises in mid-late erythroid cells and this is paralleled by loss of *LIN28B (*together with *MYC* and *IGF2BP1*). Complementing the data in Fig. 1, the expression pattern of *SOX6* and *LIN28B* during human fetal erythropoiesis at a single cell level strongly suggests a potential switch-off mechanism of *LIN28B* transcription by SOX6.

### SOX6 represses Lin28B blocking the LIN28B/Let-7 axis and cell proliferation in different erythroid cellular models

The LIN28B/Let-7 axis, which represents the main LIN28B downstream pathway, ultimately results in the control of proproliferative genes, including *MYC* and *IGF2BP1*^19^. Our observations (Figs. 1 and 2) suggest that SOX6 may interfere on the LIN28B/Let-7 axis. To test if SOX6 is causally involved in the transcriptional regulation of *LIN28B*, we overexpressed it and monitor how *LIN28B* mRNA expression is affected as a consequence. To this aim, we selected three different “fetal-like” human erythroid cells lines: K562 and HEL erythroleukemias and HUDEP1, which model human embryonic erythropoiesis (Supplementary Fig. 3).

**Figure 3:**
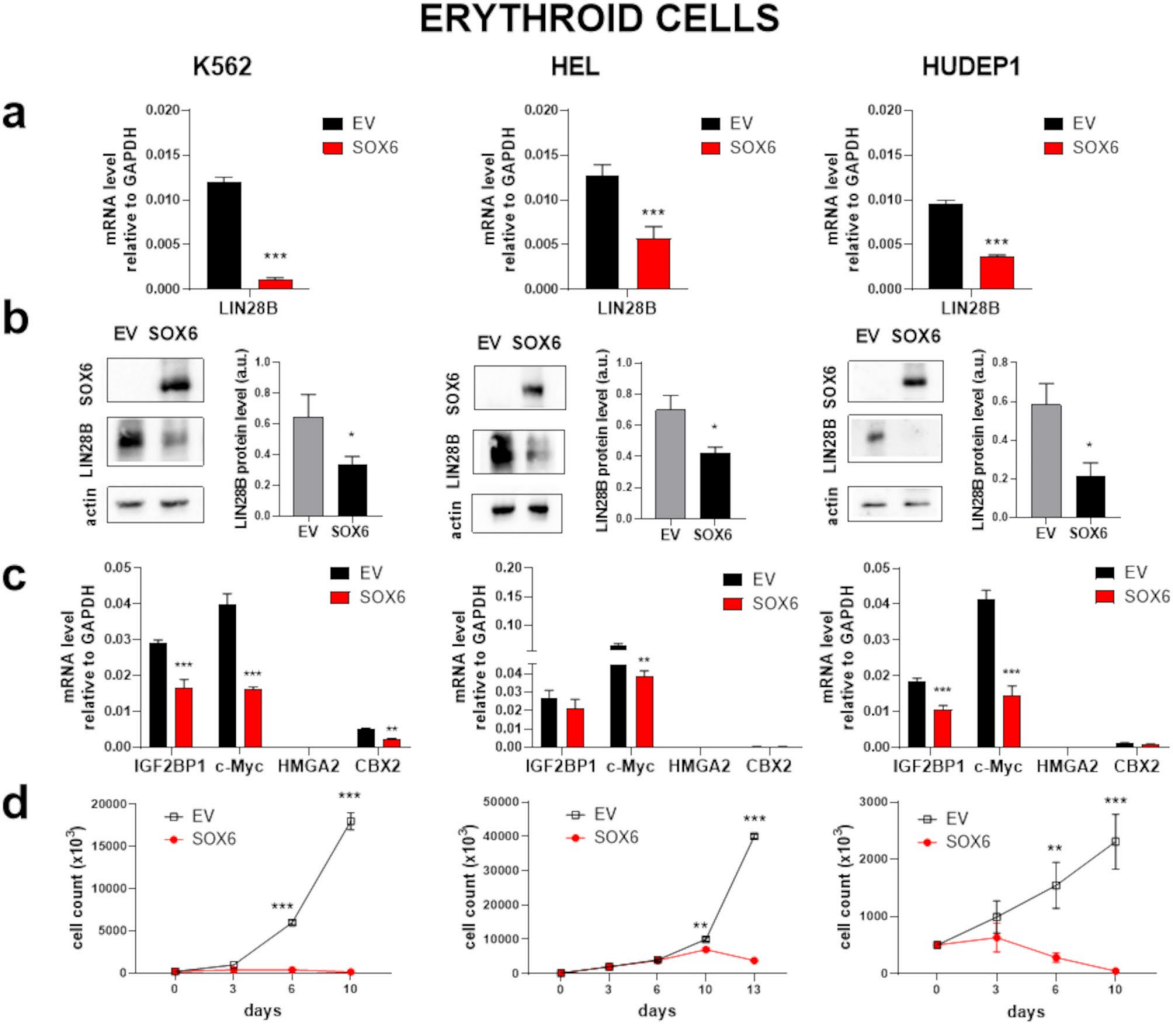
SOX6 represses *LIN28B* and blocks proliferation in erythroid cells. (**a**) Effect of SOX6 expression on *LIN28B* mRNA level. SOX6: cells transduced with the SOX6 overexpressing vector; Empty Vector (EV): cells transduced with the corresponding empty vector. Fold reduction calculated on the mean values: K562 ≈92% *p* < 0.0001, HEL ≈54% *p* = 0.0009, HUDEP1 ≈60% *p* < 0.0001. (**b**) Representative Western blots and quantification of the LIN28B reduction upon SOX6 overexpression at the protein level (densitometry analysis from 3 independent Western blot experiments was performed by using the ImageJ software). Fold reduction calculated on the mean values: K562 ≈48% *p* = 0.03, HEL ≈40% *p* = 0.0281, HUDEP1 ≈63% *p* = 0.046. (**c** and **d**) Effects of SOX6 expression on representative Let-7 downstream targets and on cell proliferation. Empty Vector (EV), in black: cells transduced with the empty vector. SOX6, in red: cells transduced with the SOX6 expressing vector. Fold reduction of targets calculated on the mean values: K562 *IGF2BP1* ≈42% *p* = 0.0005, K562 *c-MYC* ≈60% *p* < 0.0001, K562 *CBX2* ≈60% *p* = 0.0058, HEL, *c-MYC* ≈40% *p* = 0.0045. HUDEP1 *IGF2BP1*≈39% p = 0.0006, HUDEP1 *c-MYC* ≈64% p = 0.0008. For all RT-PCR shown, n ≥ 4, error bars: standard error of mean. *p < 0.05; **p < 0.01; ***p < 0.001.

We infected the above cell lines with viral pseudoparticles carrying a SOX6Flag-IRES-GFP overexpressing vector (SOX6) and, in parallel, with the corresponding empty control vector (EV), (Supplementary Fig. 1). Upon SOX6 overexpression, *LIN28B* is significantly downregulated in all the tested cell lines, both at the mRNA and at the protein level (Fig. 3a, b). In order to evaluate the secondary impact of *LIN28B* repression on the LIN28B/Let-7 axis, we selected different known Let-7 target genes (Fig. 3c), including c-*MYC*, *HMGA2, IGF2BP1* and *CBX2*^10,14,17^. Among them, *HMGA2* is not expressed in all the three cell lines, *CBX2* is expressed at low level in K562 and HUDEP1 cells and c-*MYC* and *IGF2BP1* are highly expressed in all three cell lines. IGF2BP1, which belongs to the family of the oncofetal IGF2 mRNA binding proteins, is highly expressed during embryogenesis and overexpressed in various tumors^48,49^. In erythropoiesis, IGF2BP1 is a major target of the Let-7 miRNA family^19^^50^, expressed in fetal erythroblasts^25^ and capable of inducing γ-globin expression^51,52^. As shown in Fig. 3c, *c-MYC*, *IGF2BP1* and *CBX2* (in K562) expression is reduced by SOX6 overexpression. The overexpression of SOX6 results in a marked decline in cell proliferation (Fig. 3d). We previously showed that SOX6 blocks cell proliferation in Set-2 and Uke1 myeloid cancer cells^36^. Here we show that SOX6 overexpression downregulates the LIN28B/Let-7 pathway also in these cells (Supplementary Fig. 4). Together, these data confirm the ability of SOX6 to repress *LIN28B* and the pro-proliferative genes downstream to the LIN28/Let-7 axis, pointing to a wide tumor suppressor role of SOX6.

### *LIN28B* repression by SOX6 results in the repression of Let-7 targets in non-hematopoietic LIN28B-positive cancer cells

*LIN28B* is broadly expressed in early development and is reactivated in cancers^17^. We analyzed *LIN28B* expression in the large panel of comprehensively characterized Cancer Cell Line Encyclopedia (CCLE, Broad Institute, https://sites.broadinstitute.org/ccle/). Consistently with a broad role in cancer, *LIN28B* is found highly expressed in a wide spectrum of cancer cell lines, including myeloid, liver, and central nervous system cells, compared to other tissues (Supplementary Fig. 5a). Moreover, LIN28B is overexpressed in both primary as well as metastatic cell lines (Supplementary Fig. 5c).

**Figure 4:**
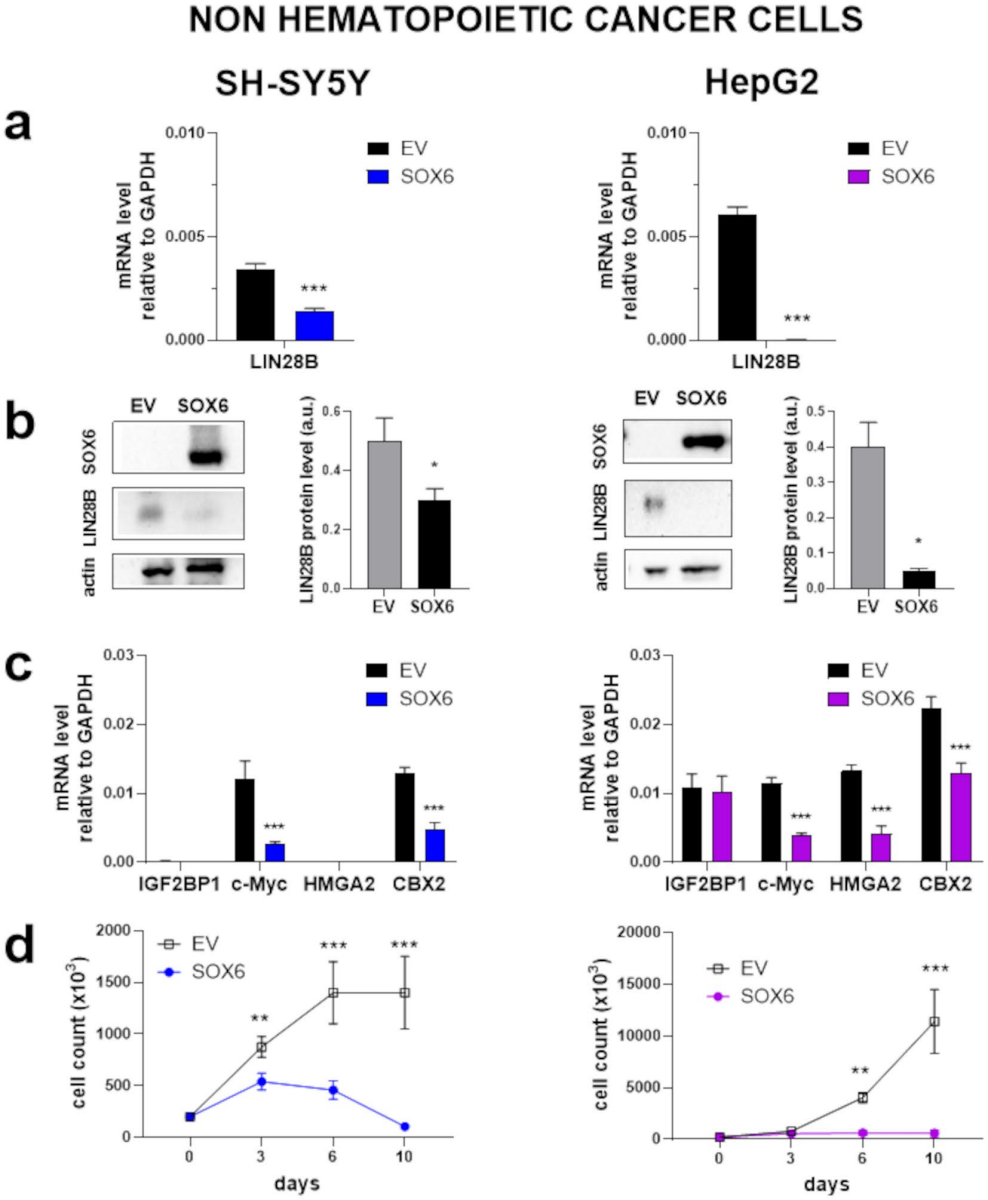
SOX6 represses *LIN28B* and blocks proliferation in neuroblastoma SH-SY5Y and hepatoblastoma HepG2 cells. The same analysis as in Fig. 3 was carried out in two non- hematopoietic cancer cells whose growth is sustained by LIN28B. SOX6: cells transduced with the SOX6 overexpressing vector; Empty Vector (EV): cells transduced with the corresponding empty vector. (**a**) mRNA fold reduction calculated on the mean values: SH-SY5Y ≈59% *p *< 0.0001, HepG2 ≈99.7% *p *< 0.0001. (**b**) Protein fold reduction calculated on the mean values: SH-SY5Y ≈40% *p *<  = 0.03 HepG2 ≈88% *p *< 0.02. (**c**) Fold reduction of targets calculated on the mean values: SH-SY5Y *c-MYC* ≈75% *p *< 0.0001, SH-SY5Y *CBX2* ≈62% *p *= 0.0004, HepG2 *c-MYC* ≈63% *p *< 0.0001, HepG2 *HMGA2*≈70% *p *= 0.0008, HepG2 *CBX2* ≈41% *p *= 0.0007. (**d**) Cell proliferation curves.

To test whether high *LIN28B* gene expression correlates with genetic dependency, we analyzed dependencies scores calculated by Chronos for *LIN28B* CRISPR in CCLE cell lines^53^. According to DepMap (https://depmap.org/portal/), *LIN28B* expression and dependency varied across cell lines. In particular, neuroblastoma cell lines, including SH-SY5Y, show high expression and significant dependency on *LIN28B* (Supplementary Fig. 5e, f). This is in line with the known involvement of LIN28B in high-risk neuroblastoma^13,54^. In addition, in neuroblastoma cell lines, SOX6 and *LIN28B* expression negatively correlate (Supplementary Fig. 5e, f). We therefore assessed the effect of SOX6 overexpression in human neuroblastoma SH-SY5Y cells (Fig. 4). To expand our observation, we performed the same analysis on human hepatic cancer HepG2 cells (Fig. 4) in which LIN28B was demonstrated by functional experiments to be essential for proliferation^11,14^.

In both cell types, we observed that overexpression of SOX6 represses *LIN28B* and ultimately results in a marked reduction of proliferation, although with slightly different kinetics. In these cells, Let-7 targets are repressed downstream to SOX6 overexpression.

Finally, we examined single-cell RNA-seq datasets obtained from neuroblastoma and hepatocellular carcinoma human biopsies (Supplementary Fig. 6). In neuroblastoma, the subset of cells expressing *SOX6* shows minimal overlap with those expressing *LIN28B*, supporting a SOX6 mediated *LIN28B* downregulation in this context (Supplementary Fig. 6a, b). In hepatocellular carcinomas, the relationship between *SOX6* and *LIN28B* expression is less clear indicating more intricate regulatory dynamics (Supplementary Fig. 6c, d).

### *LIN28B* is a direct transcriptional target of SOX6

**Figure 5:**
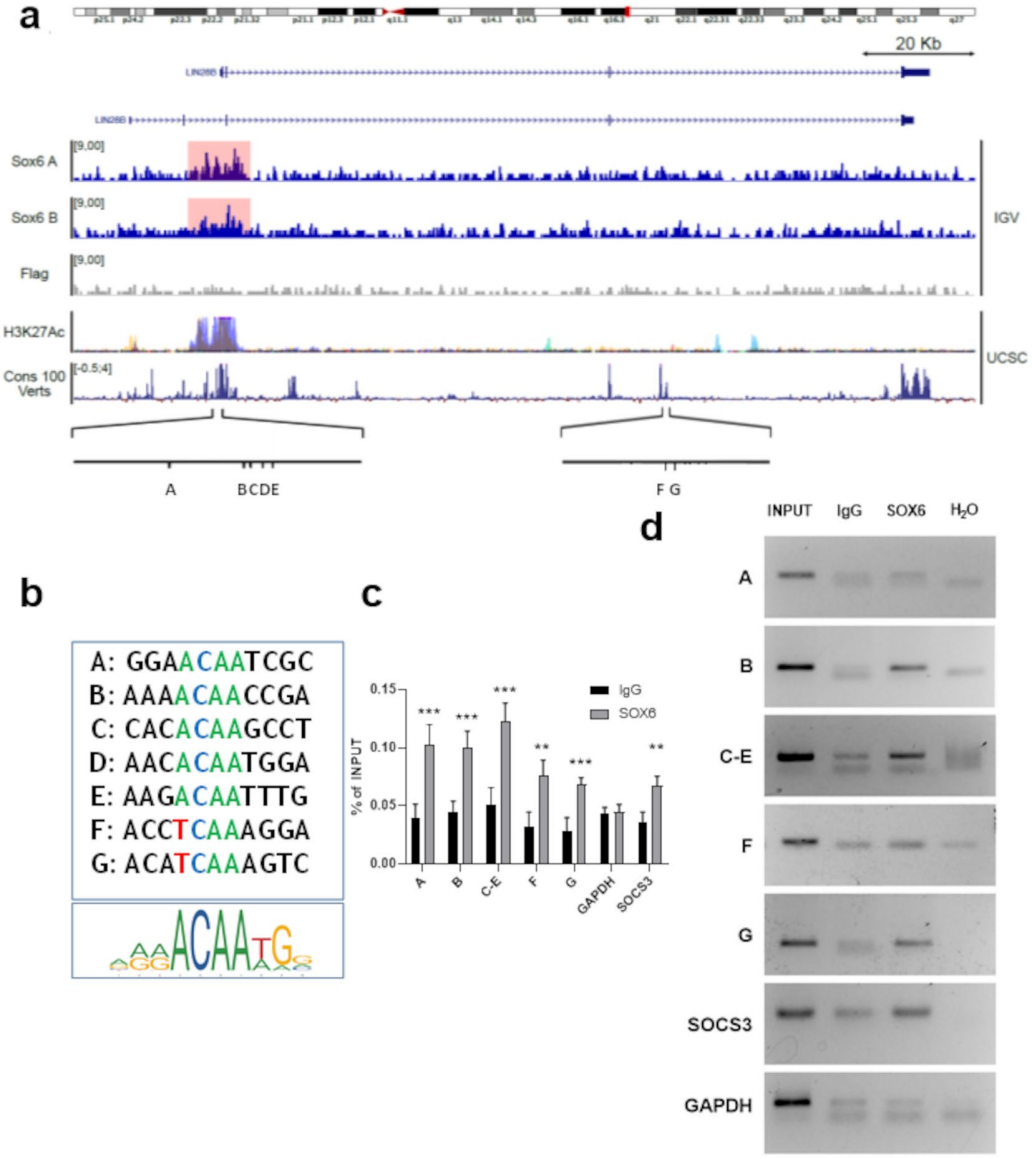
Identification of the SOX6 binding sites within the *LIN28B* locus. (**a**) *Lin28B* genomic locus visualized in Integrative Genomic Viewer (IGV) and with the UCSC genome browser (https://genome.ucsc.edu/). From the top: chromosome representation (IGV), gene transcripts (UCSC), two SOX6 CUT&RUN replicates and the respective negative Flag control in HUDEP1 cells (IGV), H3K27ac overlap (UCSC), vertebrate conservation (UCSC) and positions of the SOX6 binding sites within the promoter (A–E) and the third intron (F–G). (**b**) *SOX6* sites compared with the SOX6 consensus Jaspar matrix 515.1. (**c**) Chromatin IP results obtained in HEL cells expressing SOX6. *GAPDH* locus: negative control; *SOCS3* promoter: positive control^34^. Histograms represent the results of three biological replicates (n = 3, each of them in three technical replicates, as analyzed by RTqPCR (Error bars: standard error of mean; *p < 0.05; **p < 0.01; ***p < 0.001). (**d**) representative ChIP result. Antibodies are listed in the Supplementary table 2.

Our data points to *LIN28B* as a candidate gene transcriptionally repressed by SOX6. To test this hypothesis, we first searched for genomic binding sites of SOX6 across the *LIN28B* genomic locus. We found several SOX6 binding motifs within the *LIN28B* promoter and third intron (Fig. 5a and b, Supplementary Fig. 7). To test the ability of SOX6 to bind these sequences in vivo we performed a CUT&RUN analysis targeting the exogenously expressed SOX6-Flag in HUDEP1 (Supplementary Fig. 8). Untransduced cells were used as negative control. As shown in Fig. 5a, we identified reproducible signal enrichment in the vicinity of *LIN28B* transcriptional start site (TSS), in an evolutionarily conserved region enriched by H3K27Ac, a marker of active regulatory regions. This result constitutes robust evidence that SOX6 physically associates with this region (Fig. 5a).

Targeted chromatin immunoprecipitation (ChIP) followed by qPCR in HEL cells (Fig. 5c and d) validates the CUT&RUN findings and provides evidence for multiple binding anchors for SOX6 within the *LIN28B* locus in different cell types, suggesting robust, potentially redundant regulatory mechanisms of *LIN28B* transcription repression by SOX6.

## Discussion

*LIN28B* is a stemness-related *oncofetal* gene whose repression is required to acquire adult-specific fate in different systems^20–23,53,54^. Its reactivation is observed in several tumors^17^ and often coincide with the acquisition of fetal–like phenotypes^23,24^. Despite its broad role in development and cancer, the molecular basis of its transcriptional repression is largely unknown. In this work we identify the pro-differentiative, growth-restriction SOX6 “adult” transcription factor^31,54^ as a direct repressor of the *LIN28B* gene.

First we show that *LIN28B* and *Sox6* expression is mutually exclusive in mouse and human erythropoiesis, which represents a well-defined model of development (Figs. 1 and 2) as well in several cancer cells (Figs. 3, 4 and Supplementary Figs. 5 and 6), in particular neuroblastoma and hepatocarcinoma cell lines. SOX6 binds in vivo to several consensus binding sites that we identified within the *LIN28B* locus (Fig. 5 and Supplementary Fig. 7), represses *LIN28B* transcription and cell proliferation (Figs. 3, 4).

**Figure 6:**
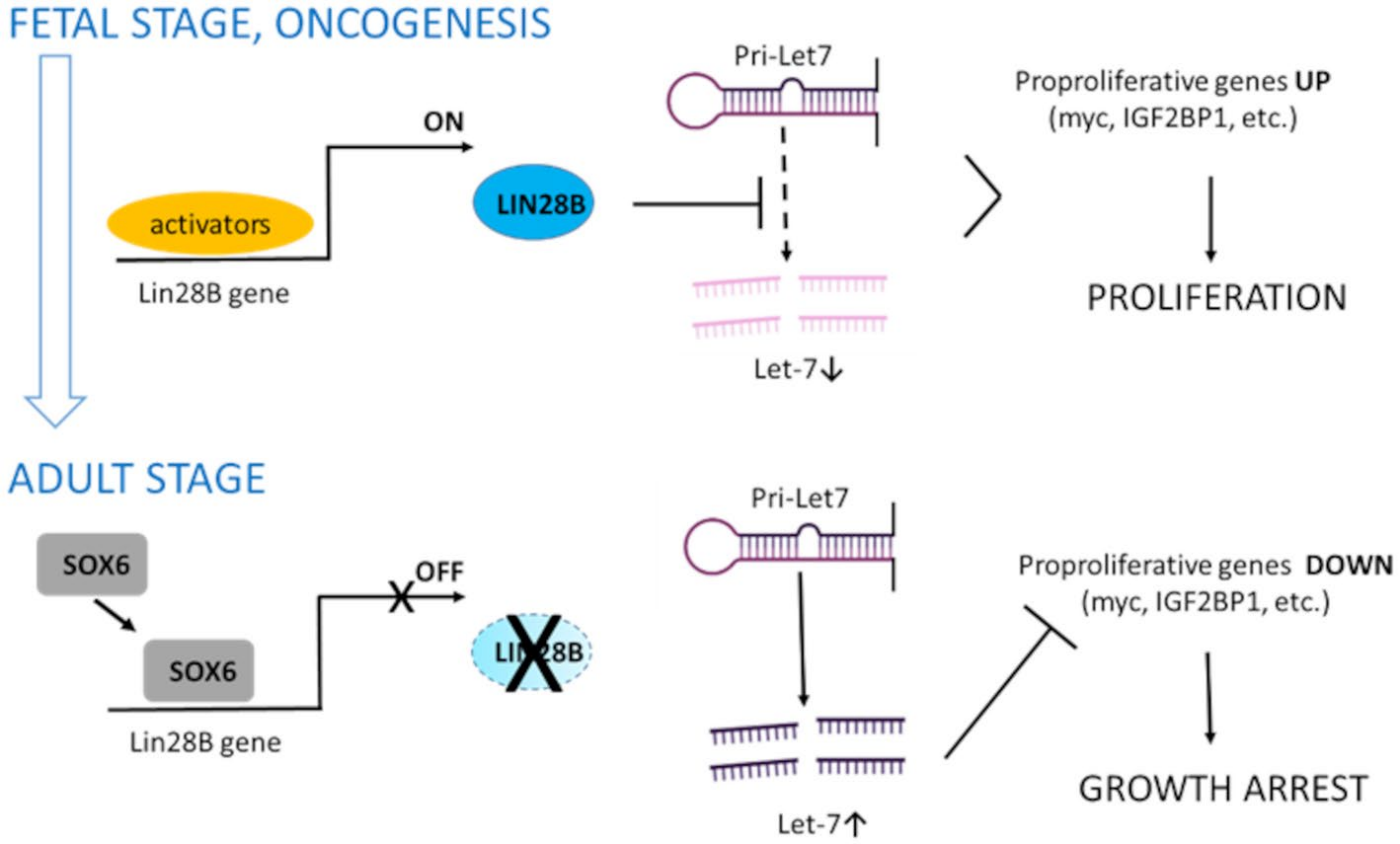
Proposed model of *LIN28B* repression by SOX6. In fetal cells and in cancers with illegitimate *LIN28B* reactivation, LIN28B blocks the processing of Let-7 precursors to Let-7 miRNA. Let-7 reduction leads to the failure of silencing of proproliferative genes, including *c-MYC* and *IGF2BP1*, thus promoting cell proliferation. SOX6, by repressing *LIN28B* transcription, allows the maturation of Let-7 miRNAs, among the most abundant miRNAs in adult cells^17^. Let-7 miRNAs post-transcriptionally repress proproliferative genes inducing growth arrest (created with Biorender).

At the molecular level, the LIN28B oncogenic potential largely relies in its ability to repress the processing of miRNAs of the Let-7 family, which in adult cells promote differentiation and repress pro-proliferative genes^17^. We demonstrate that SOX6 impacts on the expression of genes downstream to the LIN28B/Let-7 axis (Figs. 2, 3, 4), such as *c-MYC* and *IGF2BP1*, confirming the involvement of this pathway. Based on our observations, we propose a general model in which SOX6 acts as a tumor suppressor in cells of different tissue origin acting through the LIN28B/Let-7 pathway (Fig. 6).

We cannot exclude that additional molecular mechanisms/pathways downstream to SOX6, independent from *LIN28B* downregulation concur to the observed tumor suppressive outcome and it is also likely that reduction of LIN28B expression does not exclusively impact the Let-7 axis. Nevertheless, we believe that the genetic circuit involving the direct repression of *LIN28B* by SOX6 represents a novel layer of regulation with relevant therapeutic implications for those tumors relying on LIN28B for their survival, often associated with high-risk malignancy.

The identification of the mechanistic link between SOX6 and LIN28B may provide novel opportunities for tumors stratification and novel therapeutic options.

### Supplementary Information

Supplementary Information.

## Data Availability

All data have been deposited in ArrayExpress under accession number E-MTAB-12800.
