# Supplementary material for: Transcriptional repression of the oncofetal LIN28B gene by the transcription factor SOX6
Source: Sci Rep. 2024 May 4;14:10287. doi: 10.1038/s41598-024-60438-3 (PMC11069503; doi:10.1038/s41598-024-60438-3)
Supplement: Supplementary file 1 — Supplementary Information. [file 41598_2024_60438_MOESM1_ESM.pdf]

Repression of the oncofetal *LIN28B* gene by the transcription factor SOX6

Valentina Pastori, Gianluca Zambanini, Elisabetta Citterio, Tamina Weiss, Yukio Nakamura, Claudio Cantù and Antonella Ellena Ronchi

SUPPLEMENTARY FIGURES

Suppl. Fig. 1

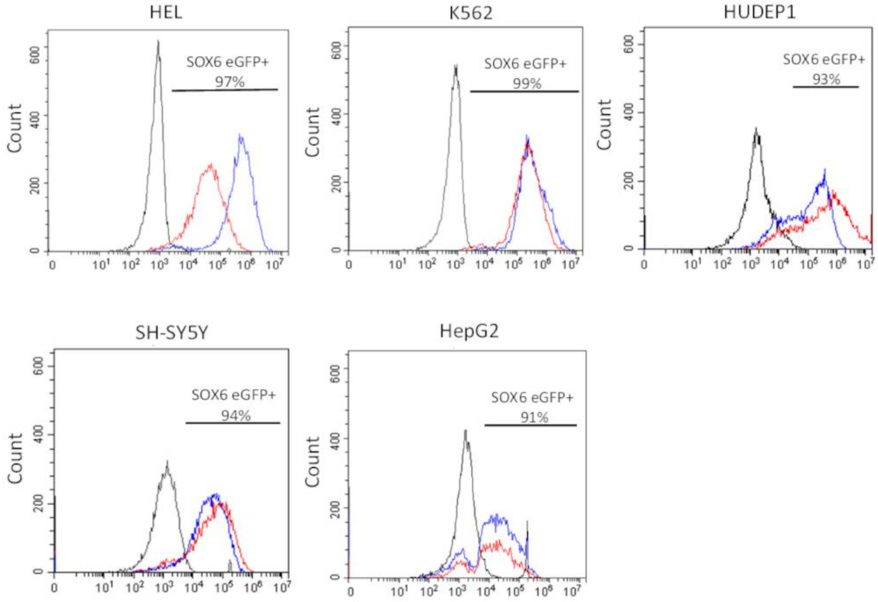

Transduction efficiency. Transduction efficiency was assessed by Flow cytometry as the CSI-SOX6 expression vector contains a bicistronic Sox6-Flag-IRES-GFP cassette[1]. x axis: Mean Fluorescence Intensity, y axis: cell count. Gray: untransduced cells. Red: GFP^+ cells infected with the SOX6 overexpressing vector. Blue: GFP^+ cells infected with the corresponding Empty Vector. HUDEP1 and HepG2 were transduced with an efficiency of about 60-70% and were then sorted to obtain the cell (>90%GFP^+) populations used (above panels).

1. Cantu, C. *et al.* Sox6 enhances erythroid differentiation in human erythroid progenitors. *Blood* **117**, 3669-79 (2011).

Suppl. Fig. 2

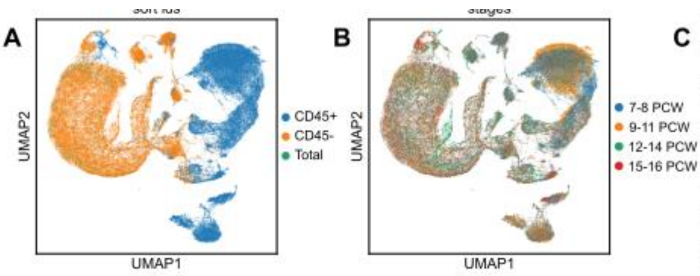

C

individual	tissue	age	stage	cell number
F2	FL	7	7-8 PCW	
F3	FL	7	7-8 PCW	
F4	FL	8	7-8 PCW	
F5	FL	8	7-8 PCW	32110
F6	FL	9	9-11 PCW	
F7	FL	9	9-11 PCW	
F8	FL	9	9-11 PCW	
F10	FL	11	9-11 PCW	34553
F11	FL	12	12-14 PCW	
F12	FL	13	12-14 PCW	
F13	FL	14	12-14 PCW	
F17	FL	12	12-14 PCW	27524
F14	FL	16	15-17 PCW	
F15	FL	16	15-17 PCW	
F16	FL	17	15-17 PCW	18876

Additional information to the human fetal liver hematopoiesis dataset (Popescu et al., 2019). a, UMAP labelled according to the fluorescence-activated cell sorting of CD45+ and – cells done prior to sequencing, indicating distinct lineages of hematopoiesis. **b,** UMAP indicating developmental stage of the derived cells. **c,** Table showing additional information about the scRNA-sequencing samples used for Figure 2 and for this figure. **d,** UMAP-blots of hemoglobin gene expression in human fetal liver (upper panels) and yolk sac (lower panels).

Suppl. Fig. 3

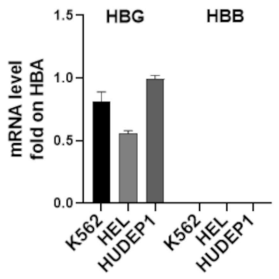

K562, Hel and HUDEP1 cell represent embryo/fetal model of erythropoiesis.
mRNA expression levels of fetal γ (HBG) and adult β (HBB) globin relative to α (HBA) globin.
For all samples, RT-qPCR n≥3, error bars: standard error of mean.

Suppl. Fig. 4

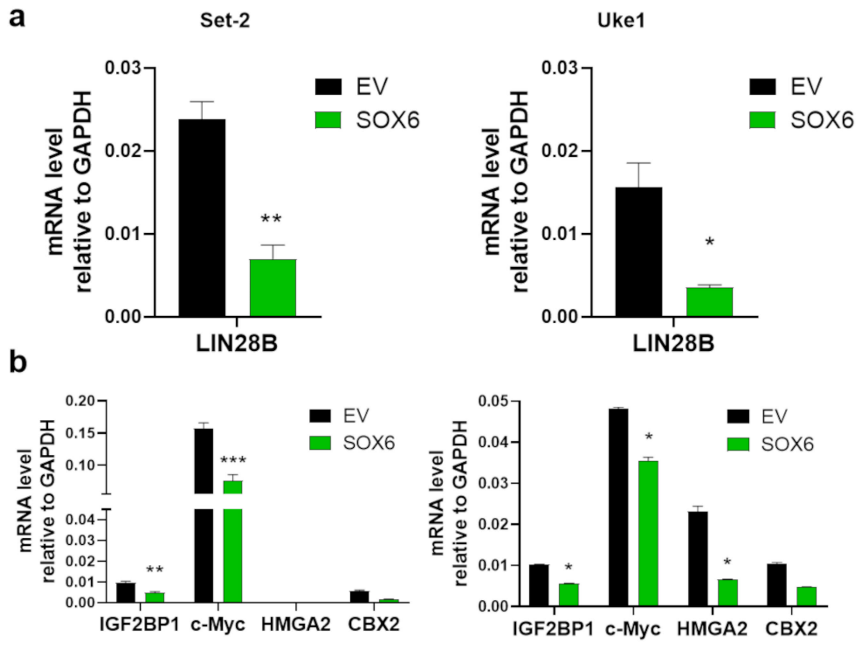

SOX6 directly represses *LIN28B* in Set-2 and Uke1 cells. Effect of SOX6 expression on *LIN28B* mRNA levels (a) and on representative Let-7 downstream targets (b).

Suppl. Fig. 5

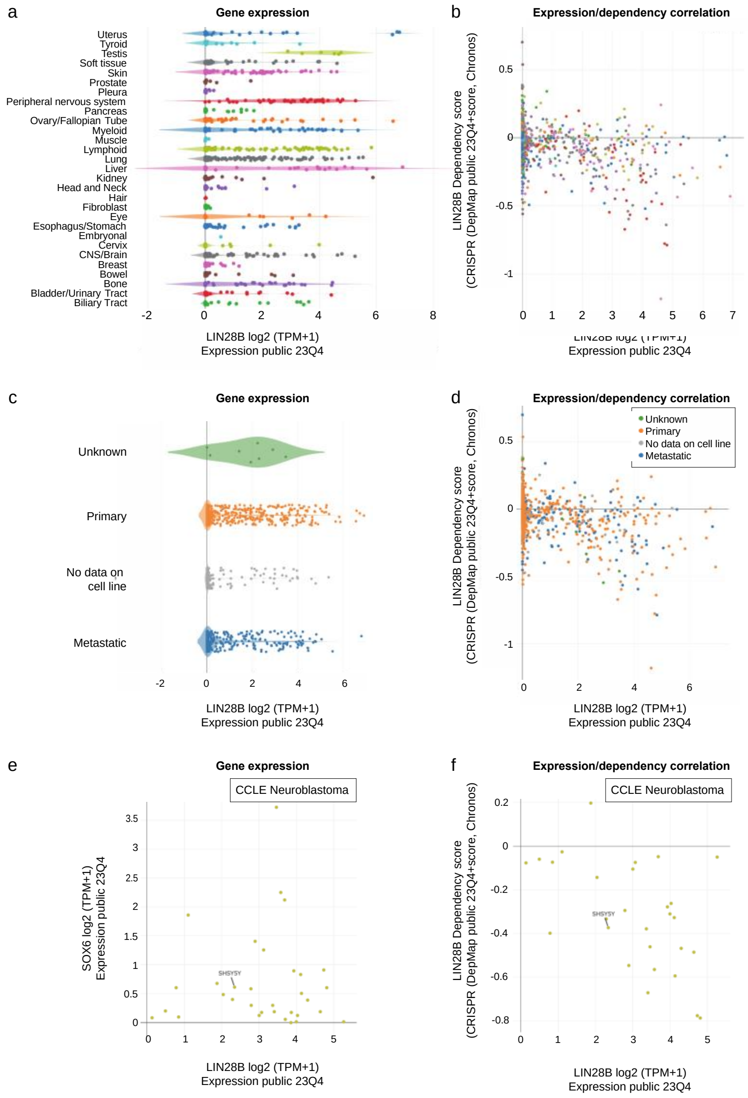

Supplemental Fig. 5

***LIN28B* gene expression in cancer.**

a, Expression of *LIN28B* from transcriptional profiling of human cancer cell lines of the indicated lineages (CCLE, Broad Institute) in DepMap. **b,** the dependency scores were calculated by Chronos (Dempster JM et al., 2021) for LIN28B CRISPR in CCLE lines shown in **a** and correlated to LIN28B gene expression levels in DepMap. **c,** Expression of *LIN28B* from transcriptional profiling of human cancer cell lines of the indicated tumor types (CCLE, Broad Institute) in DepMap. **d,** the dependency scores were calculated by Chronos. for LIN28B CRISPR in CCLE lines shown in (c) and correlated to LIN28B gene expression levels in DepMap. **e,** Expression of *LIN28B* and *SOX6* from transcriptional profiling of human cancer cell lines of the peripheral nervous system, neuroblastoma (CCLE, Broad Institute) in DepMap. **f,** the dependency scores were calculated by Chronos for LIN28B CRISPR in the CCLE neuroblastoma cell lines shown in **e** and correlated to LIN28B gene expression levels in DepMap. **a-f**: TPM (Transcripts Per Million). **b, d, f**: LIN28 Gene Effect (Chronos). CRISPR (DepMap Public 23Q4+Score, Chronos).

Suppl. Fig. 6

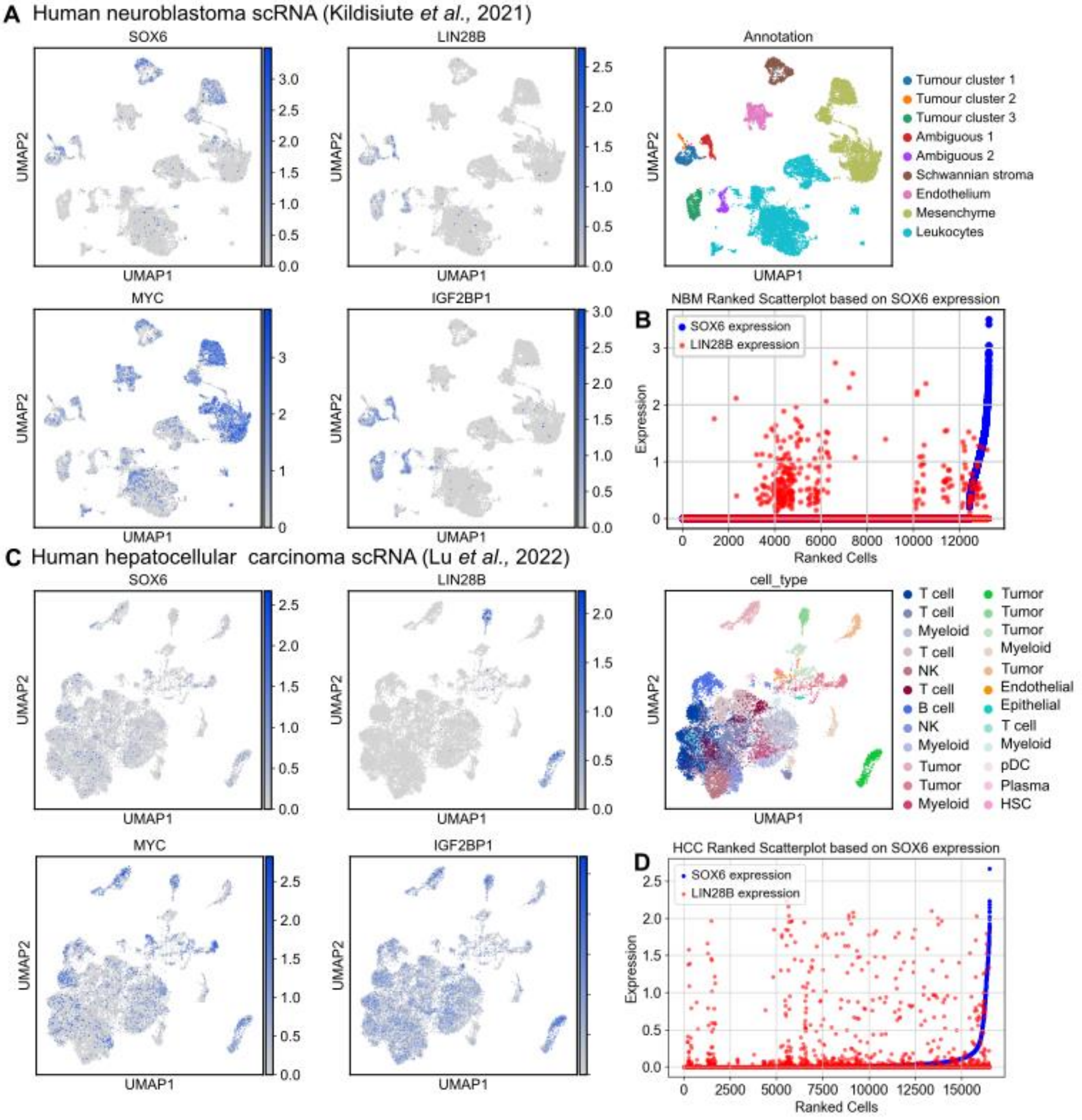

Single-cell RNA expression of SOX6, LIN28B, MYC, and IGF2BP1 in cancer.

a,b, data from Kildisiute *et al*., 2021. **a,** Single-cell RNA expression of *SOX6*, *LIN28B*, *MYC*, and *IGF2BP1* in 16498 cells derived from human neuroblastoma (NBM) biopsies. Clusters were associated with 9 cell types according to the expression of marker genes. Samples were derived from pretreated tumors biopsies, only including viable areas. **b,** ranked scatterplot sorting cells based on their expression of SOX6 along the x-axis. The y-axis indicates the normalized expression of *SOX6* (blue) and *LIN28B* (red). **c,d,** data from Lu *et al.,* 2022 **c,** single-cell RNA expression of *SOX6*, *LIN28B*, *MYC*, and *IGF2BP1* in cells derived from human hepatocellular carcinoma biopsies. Clusters were associated with 23 cell types according to the expression of marker genes. Samples were derived from primary and relapsed tumors and adjecent liver from 10 individuals. **d,** ranked scatterplot sorting cells based on their expression of *SOX6* along the x-axis. The y-axis indicates the normalized expression of *SOX6* (blue) and *LIN28B* (red).

Suppl. Fig. 7:

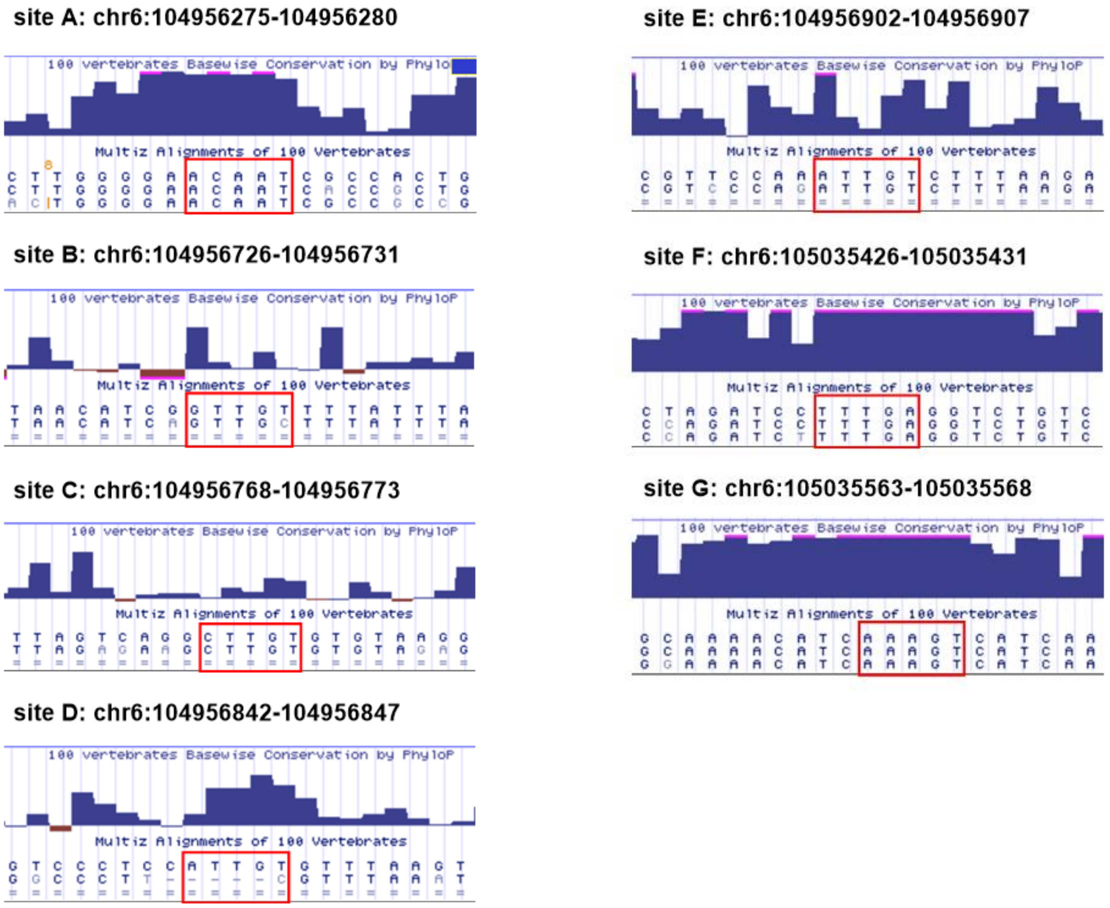

Evolutionary conservation of the SOX6 consensus binding sites within the *LIN28B* locus, according to the UCSC genome browser (https://genome.ucsc.edu/). Human, mouse and chicken sequences are shown. Coordinates refer to the human assembly GRCh38/hg38. The sites order (from A to G) refers to the Lin28B locus map in Figure 5.

Suppl. Fig. 8

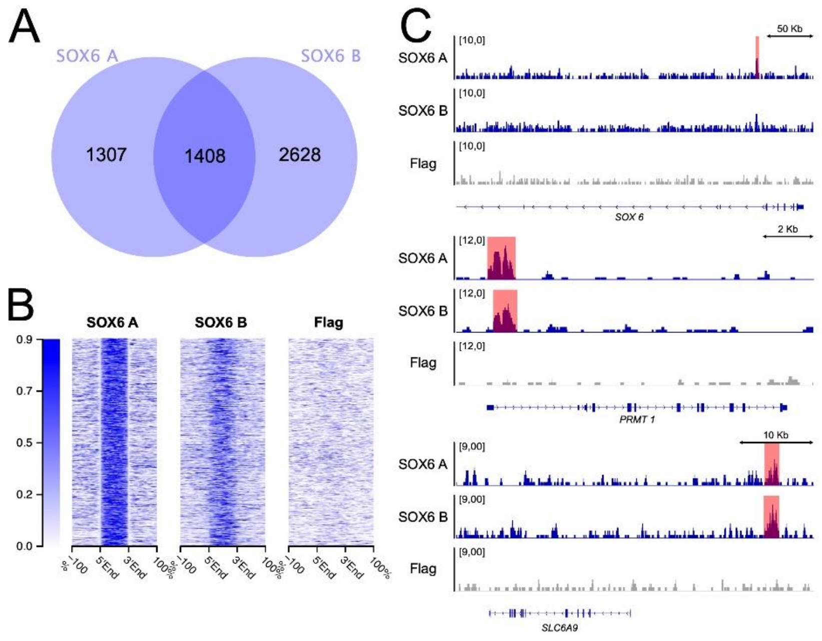

a, Venn diagram showing the number of peaks in the two replicates. The peaks present in both replicates are considered as reproducible, high-confidence events. **b,** Signal Enrichment plots for SOX6 showing common peaks from the two replicates. Signal entries in the heatmap are ordered by overall enrichment of the first profile on the left. **c**, Genomic coverage tracks obtained with CUT&RUN LoV-U targeting SOX6 representing the *SOX6*, *PRMT1* and *SLC6A9* loci and visualized with Integrative Genomic Viewer (IGV). From the top: two SOX6 replicates (SOX6 A and SOX6 B) with their respective negative control (anti-Flag).

Suppl. Fig. 9

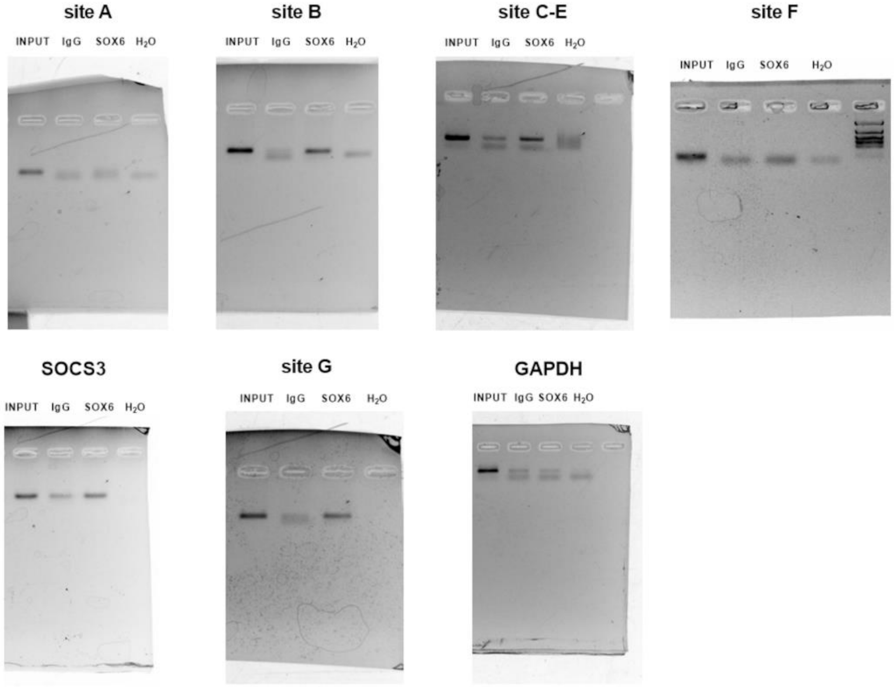

Uncropped gels corresponding to panel d of Figure 5 (Chromatin Immunoprecipitation)

Suppl. Fig. 10

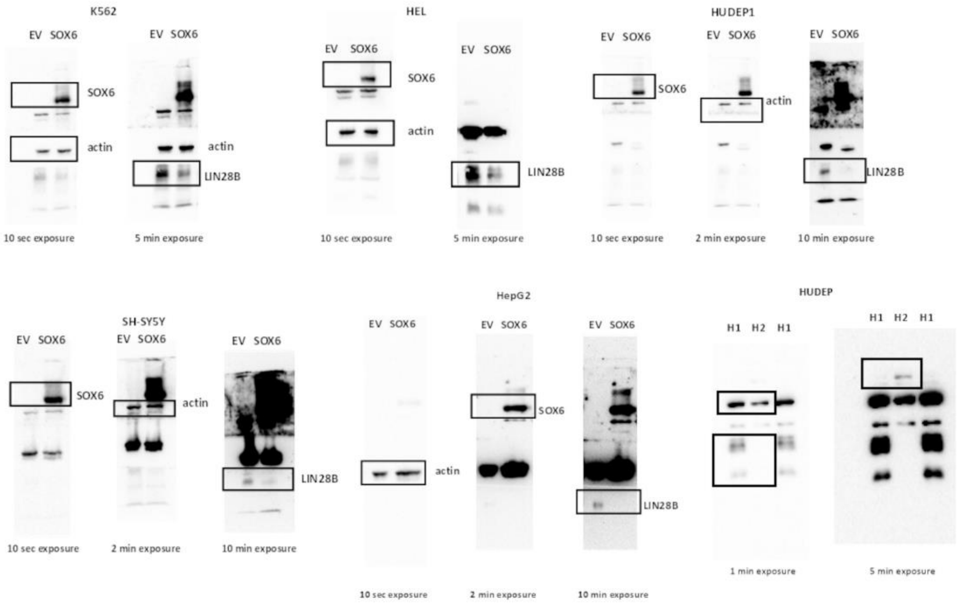

Uncropped Western blots filters corresponding to Figures 1, 3-4. Within boxes, the selected regions.

SUPPLEMENTARY MATERIAL

Supplementary Table 1: Cell culture media composition and supplements

Cell line	MEDIUM	Supplements
**K562**	RPMI	Fetal Bovine Serum 10%
**Hel**	RPMI	Fetal Bovine Serum 10%
**Set-02**	RPMI	Fetal Bovine Serum 20%
**Uke1**	IMDM	Fetal Bovine Serum 10%
**HepG2**	DMEM	Fetal Bovine Serum 10%
**SH-SY5Y**	DMEM	Fetal Bovine Serum 10%
**HUDEP1[1]**	IMDM	0.2% HSA (Human Serum Albumin); 0.1% Lipid Mixture; 300 µg/mL transferrin; 10 µg/mL insulin; 100 µM sodium pyruvate; 2 U/mL EPO (Erythropoietin); 100 ng/mL SCF (Stem Cell Factor); 1 µM dexamethasone; 2 µg/mL doxycycline.

[1]Kurita, R. et al. Establishment of immortalized human erythroid progenitor cell lines able to produce enucleated red blood cells. PLoS One 8, e59890 (2013).

Supplementary Table 2: list of antibodies and reagents used in this study

Antibodies and reagents	Cat n°	Manufacturer
**Anti-FLAG (ChIP)**	F7425	Sigma-Aldrich
**Anti-LIN28B human**	4196	Cell Signaling
**Anti-LIN28B mouse**	BK5422	Cell Signaling
**Anti-actin**	47778	Santa Cruz
**HRP-conjugated anti-rabbit IgG**	7074	Cell Signaling
**Anti-SOX6**	ab30455	Abcam
**Anti-SOX6 (Western blot)**	AB5805	Millipore
**rIgG**	PP64	Millipore
**DMEM medium**	ECB7501L	Euroclone
**RPMI Medium 1640**	ECB9006L	Euroclone
**Phosphate-buffered saline (PBS)**	ECB4004L	Euroclone
**Fetal Bovine Serum (FBS)**	F7524	Sigma-Aldrich
**L-glutamine**	ECB3000D	Euroclone
**Penicillin-Streptomycin**	ECB3001D	Euroclone
**Luminata Western HRP substrate**	WBLUR0500	Millipore
**High Capacity cDNA RT Kit**	4368814	Applied Biosystems
**Trypsin-EDTA**	ECB3052D	Euroclone
**SsoAdvanced Universal SYBR® Green**	1725274	Bio-Rad
**IMDM medium**	P04-20250	PAN
**HUMAN SERUM ALBUMIN**	P06-27100	PAN
**rh SCF**	11343325	ImmunoTools
**rh EPO**	11344795	ImmunoTools
**dexamethasone**	D4902	Sigma-Aldrich
**doxycicline**	D9891	Sigma-Aldrich

Supplementary Table 3: list of primers used in RT-PCR and ChIP experiments
(A-G: human Lin28B locus)

**Primers used for RT-PCR**		
**GENE**	**F/R**	**Sequence (5'-3')**
**h_LIN28B**	**F**	GCGCATGGGATTTGGATTCA
	**R**	ACTTCCTAAACAGGGGCTCC
**m_LIN28B**	**F**	ACGGCAGGATTTACTGATGG
	**R**	GCACTTCTTTGGCTGAGGAG
**m_LIN28A**	**F**	AAGCGCAGATCAAAAGGAGA
	**R**	CTGATGCTCTGGCAGAAGTG
**h_HMGA2**	**F**	ACCCAGGGGAAGACCCAAA
	**R**	CCTCTTGGCCGTTTTTCTCCA
**h_IGF2BP1**	**F**	TAGTACCAAGAGACCAGACCC
	**R**	GATTTCTGCCCGTTGTTGTC
**m_GAPDH**	**F**	ACCACAGTCCATGCCATCAC
	**R**	TCCACCACCCTGTTGCTGTA
**h_GAPDH**	**F**	ACGGATTTGGTCGTATTGGG
	**R**	TGATTTTGGAGGGATCTCGC
**HBA**	**F**	GAGGCCCTGGAGAGGATGTTCC
	**R**	ACAGCGCGTTGGGCATGTCGTC
**HBB**	**F**	TACATTTGCTTCTGACACAAC
	**R**	ACAGATCCCCAAAGGAC
**HBG**	**F**	CTTCAAGCTCCTGGGAAATGT
	**R**	GCAGAATAAAGCCTATC TTGAAAG
**h_SOX6**	**F**	GAGGCAGTTCTTTACTGTGG
	**R**	CCGCCATCTGTCTTCATA
**m_SOX6**	**F**	TTCCTCCTGCATGGAAAAAC
	**R**	GATGCTGCCAGCTTTTTCTG
**h_c-myc**	**F**	CCTTGCAGCTGCTTAGACG
	**R**	AAGTTCTCCTCCTCGTCGC
**h_CBX2**	**F**	GACAGAACCCGTCAGTGTCC
	**R**	GGCTTCAGTAATGCCTCAGGT

Primers used in Chromatin Immunoprecipitation experiments
(A-G: human Lin28B locus)

**site A**	**F**	CCTGCCCTATGTCCTCC
	**R**	CCATCTCTTTCCGGGGG
**site B**	**F**	GTTTCATGGGGATGTAATTAGG
	**R**	CTAAAATCATGGGAGGGGGTCG
**site C-E**	**F**	CCTCCCATGATTTTAGTCAGGC
	**R**	CGCCATTTCATCTATGCTGACA
**site F**	**F**	GGAATTTGCTGTTTAGTAGG
	**R**	CCTGCCCTCAGATGTTGACC
**site G**	**F**	GGCAGTTTAAAGACCAG
	**R**	CTGATTCCAACAAACAC
**GAPDH**	**F**	CGGAGTCAACGGATTTGGTCGTAT
	**R**	AGCCTTCTCCATGGTGGTGAAGAC
**SOCS3**	**F**	CTC TCG GCA GAG GTT TAT GG
	**R**	TCA AGG AAT AGC CCT TGA GG

CUT&RUN (LoV-U)

CUT&RUN was performed according to the LoV-U protocol described in (Zambanini et al., 2022). Per each sample 500,000 HUDEP1 (SOX6-Flag expressing) were harvested. Cells were washed three times in Nuclear Extraction (NE) buffer (HEPES-KOH pH-8.2 [20 mM], KCl [10 mM], Spermidine [0.5 mM], IGEPAL [0.05%], Glycerol [20%], Roche Complete Protease Inhibitor EDTA-Free), then resuspended in 40 µl NE per sample and bound to 20 µl Magnetic ConA Agarose beads (ABIN6952467) equilibrated in binding buffer (HEPES pH-7.5 [20 mM], KCl [10 mM], CaCl2 [1 mM], MnCl2 [1 mM]). After incubation, nuclei and beads were resuspended for 5 minutes in EDTA wash buffer (HEPES pH-7.5 [20 mM], NaCl [150 mM], spermidine [0.5 mM] in Roche Complete Protease Inhibitor EDTA-Free (COEDTAFRO), + EDTA [0.2 mM]). Samples were divided in 200 µl PCR tubes and antibody incubation was performed in 200 µl wash buffer with 2 µl of anti-FLAG antibody (Cat. F7425, Sigma-aldrich) overnight (ON) at 4 °C on a rotator. After ON incubation, samples were washed five times in wash buffer and resuspended in 200 µl of pAG-MN buffer (wash buffer supplemented with pAG-MN [0.6 µg/ml]) for 30 minutes at 4 °C on a rotator.pAG/MNase was a gift from Steven Henikoff (Addgene #123461), expressed and purified as described previously (Meers et al., 2019a). Samples were washed five times, followed by digestion for 30 minutes in wet ice in 50 µl wash buffer with 2 mM CaCl2. After 30 minutes, the digestion buffer was moved to new collection tubes and the reaction on the beads was stopped with 50 µl of 1X Urea STOP buffer (NaCl [100 mM], EDTA [2 mM], EGTA [2 mM], IGEPAL [0.5%], Urea [8.5 M]) and the samples were incubated for 1 hour at 4°C. Beads were collected on the magnetic rack, while the supernatant was mixed in the corresponding collection tube, where it was cleaned up twice using Mag-Bind TotalPure NGS beads (Cat. #M1327, Omega Bio- Tek) at 2X, and then resuspended in 20 µl Tris-HCl pH 7.5.

Library preparation and sequencing

Library preparation was performed using the KAPA Hyper Prep Kit for Illumina platforms (Cat. #KK8504, KAPA Biosystems) according to the manufacturer's guidelines with the following modifications. End repair and A-tailing was performed with 0.4 size reactions with 20 µl of purified DNA. The thermocycler conditions were set to 12 °C for 15 min, 37 °C for 15 min and 58 °C for 25 min to prevent thermal degradation of the shortest fragments. Adapter ligation was done with 0.4 size reactions. KAPA Dual Indexed adapters were used at 0.15 µM. A post-ligation clean-up was performed with Mag-Bind TotalPure NGS beads at 1.2X. Resuspension was done in 10 mM Tris-HCl pH 8.0. Library amplification was performed with 0.5 size reactions. The cycling conditions were set as follows: 45 sec initial denaturation at 98 °C, 15 sec denaturation at 98 °C, 10 sec annealing/elongation at 60 °C, 1 min final extension at 72 °C, hold at 4 °C, with 13 cycles. After amplification, a post-amplification cleanup was performed with 1.2X beads. Libraries were then run on an E-Gel EX 2% agarose gel (Cat. # G402022, Invitrogen) for 10 min using the E-Gel Power Snap Electrophoresis System (Invitrogen). Bands of interest between 150 and 500 bp were cut out and purified using the QIAquick Gel Extraction Kit (Cat. #28706, QIAGEN) according to manufacturer's instructions. Libraries were quantified with the Qubit (Thermo Scientific) using their high sensitivity DNA kit (Cat #Q32854, Thermo Scientific), pooled and sequenced 36 bp pair-end on the NextSeq 550 (Illumina) using the Illumina NextSeq 500/550 High Output Kit v2.5 (75 cycles) (Cat. #20024906, Illumina). The CUT&RUN datasets (raw and processed files) have been deposited at ArrayExpress (https://www.ebi.ac.uk/arrayexpress/) under accession number E-MTAB-12800.

Data analysis

Trimming was performed using bbmap bbduk (Bushnell *et al*, 2017, version 39.0) removing adapters, artifacts, poly AT, G and C repeats. Reads were aligned to the hg38 genome with bowtie (Langmead *et al*, 2009, version 1.3.1) using options -v 0 -m 1 -X 500. Samtools (Li et al., 2009), version 1.6) view, fixmate, markdup and sort were used to create bam files, mark and remove duplicates, and sort bam files. Mitochondrial reads were removed together with the Blacklist of problematic regions as described in Nordin et Al 2022). Individual track bedgraphs were created using bedtools (Quinlan & Hall, 2010, version 2.30.0) genomecov on pair-end mode. Peaks were called using SEACR (MEERS et al, version 1.3) against the corresponding negative control using the options "norm, relaxed". Venn diagrams and overlap peak sets were created using Intervene (Khan & Mathelier, 2017, version 0.6.5). Motif analysis was done using Homer (Heinz *et al*, 2010, version 4.11) findMotifsGenome to find motifs in the hg38 genome using -size given. Peak set gene annotation was done using GREAT (McLean *et al*, 2010, version 4.0.4) with default parameters. Signal intensity plots were created using ngsplot (Shen *et al*, 2014, version 2.63) with options -N 2 -GO total -SC global.

1.Gianluca Zambanini, Anna Nordin, Mattias Jonasson, Pierfrancesco Pagella, Claudio Cantù. A new CUT&RUN low volume-urea (LoV-U) protocol optimized for transcriptional co-factors uncovers Wnt/β-catenin tissue-specific genomic targets. *Development* 1 December 2022; 149 (23): dev201124. doi: https://doi.org/10.1242/dev.201124
2.Brandine, G.D.S., and Smith, A.D. (2022). Falco: high-speed FastQC emulation for quality control of sequencing data [version 2; peer review: 2 approved]
3.Bushnell, B., Rood, J., and Singer, E. (2017). BBMerge – Accurate paired shotgun read merging via overlap. 1–15.
4.Langmead, B., Trapnell, C., Pop, M., and Salzberg, S.L. (2009). Ultrafast and memory-efficient alignment of short DNA sequences to the human genome. *10*.

5.Li, H., Handsaker, B., Wysoker, A., Fennell, T., Ruan, J., Homer, N., Marth, G., Abecasis, G., and Durbin, R. (2009). The Sequence Alignment/Map format and SAMtools. Bioinformatics *25*, 2078–2079.
6.Quinlan, A.R., and Hall, I.M. (2010). BEDTools: A flexible suite of utilities for comparing genomic features. Bioinformatics *26*, 841–842.

7.Anna Nordin, Gianluca Zambanini, Pierfrancesco Pagella, Claudio Cantù
bioRxiv 2022.11.11.516118; doi: https://doi.org/10.1101/2022.11.11.516118
8.Meers, M.P., Tenenbaum, D. & Henikoff, S. Peak calling by Sparse Enrichment Analysis for CUT&RUN chromatin profiling. *Epigenetics & Chromatin* **12**, 42 (2019). https://doi.org/10.1186/s13072-019-0287-4
9.Khan, A., and Mathelier, A. (2017). Intervene: A tool for intersection and visualization of multiple gene or genomic region sets. BMC Bioinformatics *18*, 1–8.
10.Heinz, S., Benner, C., Spann, N., Bertolino, E., Lin, Y.C., Laslo, P., Cheng, J.X., Murre, C., Singh, H., and Glass, C.K. (2010). Simple Combinations of Lineage-Determining Transcription Factors Prime cis - Regulatory Elements Required for Macrophage and B Cell Identities. Mol. Cell *38*, 576–589.
11.McLean, C.Y., Bristor, D., Hiller, M., Clarke, S.L., Schaar, B.T., Lowe, C.B., Wenger, A.M., and Bejerano, G. (2010). GREAT improves functional interpretation of cis-regulatory regions. Nat. Biotechnol. *28*, 495–501.
12.Shen, L., Shao, N., Liu, X., and Nestler, E. (2014). ngs.plot : Quick mining and visualization of next-generation sequencing data by integrating genomic databases. 1–14.
